# Reproduction of the Inferior Maxillary Bone

**Published:** 1853-10

**Authors:** 


*Reproduction of the Inferior Maxilary Bone.*—Dr. Jones, of Kintre, Ohio, gives an interesting case of this kind, in the American Journal of Dental Science, see July No., '53, pp. 592. The case was a lady, thirty-five years of age, who lost her inferior maxilary bone, by severe salivation. The entire bone, as far back as the cœronoid processes, became necrosed, and singularly enough, was kept in the mouth with all its offensiveness for several years, five to eight—without being removed—after nature had effected its removal, she wore for several years, a "roll of linnen or cotton rags, of sufficient size and firmness, to keep the chin at a suitable degree of forward projection; rising high enough to keep the lip from falling in, and the tongue from protruding out of the mouth." In the mean time, a new deposit of bone, appears to have taken place; commencing at the chin.

Dr. Jones remarks, that "from all I was able to learn, in reference to it, I thought, and continue to regard it, a genuine reproduction of the inferior maxillary bone, so far as affected; and further, that "it does not appear to have been the spurious offspring of a hot and hasty freak of nature, but the legitimate produce of a *cool, deliberate, well intentioned resolve* and purpose, pertinaciously adhered to, untill accomplished, though by no means begun in a hurry, as the history of the case evidently shows ; and it was cleverly and handsomely done, confering on the sufferer an instrument of great usefulness, while it did much to remedy her deformity, and make her life not only tolerable but desirable."

Such singular cases hardly ever come singly, but like bad or good luck, come in clusters.

In the April No. of the Register, vol. 6, we gave a case of partial reproduction, of the inferior maxillary bone, reported by Dr. Todd, of Monongahela city, Pa. This case would appear to controvert the position assumed by Prof. C. A. Harris, that the "loss of the alveoli, simply, whether occasioned by mechanical violence, necrosis, or any other cause, are ever replaced," for in this case the teeth are also being reproduced.

